# Chronic noise exposure induces Alzheimer’s disease-like neuropathology and cognitive impairment via ferroptosis in rat hippocampus

**DOI:** 10.1265/ehpm.24-00126

**Published:** 2024-09-28

**Authors:** Jialao Ma, Jinwei Zhang, Zejin Ou, Yixian Ren, Kangyong Wu, Yifan Zhang, Siran Chen, Zhi Wang

**Affiliations:** 1 The Affiliated Guangzhou Twelfth People's Hospital, Guangzhou Medical University, Guangzhou 510620, China; 2 Key Laboratory of Occupational Environment and Health, Guangzhou Twelfth People’s Hospital, Guangzhou 510620, China; 3 School of Basic Medicine and Public Health, Jinan University, Guangzhou 510632, China

**Keywords:** Noise, Alzheimer’s disease, Rarres2, Mendelian randomization, Ferroptosis

## Abstract

**Background:**

Chronic noise exposure poses a remarkable public health concern, drawing attention to its impacts on the brain. Ferroptosis is involved in several brain-related diseases. However, the role of ferroptosis in the effects of chronic noise on the brain remains elusive. This study aimed to investigate the effects of chronic noise exposure on the brain and elucidate the underlying mechanisms.

**Methods:**

A chronic noise-induced cognitive impairment model in rats was constructed and validated. The pathological state and ferroptosis level of the rat hippocampus were determined using Western blotting and immunohistochemistry. Bioinformatics was employed to investigate the interrelationship between chronic noise exposure and genes. Genetic relationships were analyzed using Mendelian randomization (MR) analysis. Cytoscape was employed for the prediction of upstream molecular and drug targets.

**Results:**

*In vivo* experiments revealed that chronic noise exposure could induce Alzheimer’s disease (AD)-like neuropathological changes in rat hippocampus and cognitive impairment. Moreover, protein markers indicative of ferroptosis and levels of lipid peroxidation were quantified to elucidate underlying mechanisms. Thereafter, oxidative stress- and ferroptosis-related differentially expressed genes (DEGs) underwent functional enrichment and PPI network analyses. Additionally, 8 genes with diagnostic significance were identified. In MR analysis, retinoic acid receptor responder 2 (*Rarres2*) gene exhibited a negative genetic relationship with AD.

**Conclusions:**

Chronic noise exposure could induce AD-like neuropathological changes and cognitive impairment via ferroptosis. The results of MR analysis indicated that *Rarres2* gene may act as a protective factor in AD. This gene may be upstream of ferroptosis and serve as a target for the prevention and treatment of chronic noise-induced cognitive impairment.

**Supplementary information:**

The online version contains supplementary material available at https://doi.org/10.1265/ehpm.24-00126.

## 1. Introduction

Chronic exposure to environmental noise, prevalent in urban and industrial settings, has emerged as a significant public health concern [1–4]. The adverse effects of noise extend beyond those affecting hearing. Increasingly, studies have associated chronic noise exposure with a spectrum of systemic health issues, including cardiovascular diseases and mental health disorders. Furthermore, the potential of noise pollution to influence neurological health, particularly in the context of neurodegenerative diseases such as Alzheimer’s disease (AD), has attracted the attention of researchers [5–7].

A substantial body of evidence, both epidemiological and experimental, supports the hypothesis that environmental stressors such as noise may activate or intensify neurodegenerative pathways [8]. In 2021, a World Health Organization report made clear the correlation between noise exposure and cognitive impairment [9]. A cross-sectional study conducted in a Chinese shipyard examined the cognitive abilities of 563 noise-exposed workers with normal hearing. It was found that workers exposed to high-level noise (>90 dB(A)) scored lower on the test than those exposed to low-level noise [10]. Moreover, *in vivo* studies have shown that noise exposure can induce AD-like neuropathological changes in the brain, characterized by the accumulation of amyloid-beta (Aβ), Tau hyperphosphorylation, and extensive neuronal damage [11].

Although it is clear that chronic noise exposure may hasten the onset of AD, the precise mechanisms underlying this influence remain to be fully elucidated. A particularly promising area of investigation is the role of ferroptosis, a type of iron-dependent, regulated cell death marked by excessive lipid peroxidation [12, 13]. Ferroptosis has been linked to a number of neurological disorders, including AD, Parkinson’s disease, and Huntington’s chorea [14–16]. Nevertheless, the relationship between chronic noise exposure, ferroptosis and AD-like neuropathology remains largely unexplored. This cell death pathway offers a novel perspective on the impact of environmental stressors, such as noise, on neurodegenerative processes. This suggests that metabolic and oxidative imbalances in the brain, particularly in regions susceptible to AD pathology, such as the hippocampus, may be accelerated by such stressors. The hippocampus is of critical importance for the functions of learning and memory.

Therefore, the present study aims to elucidate the potential mechanistic link between chronic noise exposure, ferroptosis, and the development of AD-like neuropathology and cognitive impairment in the rat hippocampus. To mitigate bias stemming from age-related cognitive decline, Wistar rats were utilized for modeling purposes in this study [17]. Subsequently, the involvement of ferroptosis in chronic noise-induced cognitive impairment was explored through experimental and bioinformatics analyses. Furthermore, Mendelian randomization (MR) analysis was employed to analyze the relationship between ferroptosis-related genes (retinoic acid receptor responder 2, *Rarres2*) and cognitive impairment. MR is an analytical method that utilizes single-nucleotide polymorphisms (SNPs) to explore causal relationships between exposure and outcome variables [18, 19]. The study flowchart is illustrated in Supplementary Fig. 1. The multifaceted approach of this study not only contributes to a deeper understanding of the impact of chronic noise on neurological health, but may also reveal new preventive and therapeutic strategies for neurodegenerative disorders with important public health implications.

## 2. Materials and methods

### 2.1 Animals and noise exposure

A total of 24 male Wistar rats (weight, 200–220 g; age, 6 weeks) were purchased from the Animal Experiment Center of Southern Medical University (Guangzhou, China), quarantined at Guangzhou Huateng Biomedical Science and Technology Co. Ltd., and entered into the rearing room after observing no abnormality for 6 days (Laboratory Animal Use License: SYXK YUE, 2023-03-07). Rats were randomly divided into two groups: control (CON) and noise-exposed (NE), with 12 rats in each group. They were housed in rearing cages, three rats per cage. For the noise exposure, two specially prepared boxes were used, each accommodating two cages of rats.

Chronic noise exposure refers to prolonged or repeated exposure to high levels of noise over an extended period, which can lead to adverse health effects. In this study, the noise exposure protocol was based on methodologies outlined in previous studies [17, 20]. Rats in the NE group were continuously exposed to white noise at 100 dB SPL for 4 hours each day over a period of 30 days. To ensure the stability of the noise exposure, the sound level in the noise-exposed box was measured using a sound level meter with a maximum error of 1 dB. Rats in the CON group received the same treatment, except for the noise exposure.

With the exception of the 4 h of daily noise exposure (8 a.m. to 12 a.m., CON group was similarly treated but had no noise exposure), the rest of the day was feeding and relaxing time for both groups of rats, which were housed in a specific pathogen free (SPF) environment with controlled ambient temperature of 21–25 °C, controlled ambient humidity of 40–70%, and a light/dark cycle of 12 h. Food and water were available free of charge.

### 2.2 Morris water maze

The MWM test was employed to evaluate the learning and memory abilities of rats following 30 days of noise exposure [21]. The MWM consisted of a circular pool measuring 50 cm in height and 180 cm in diameter. Opaque water was maintained at a controlled depth of 30 cm and a temperature of 22 ± 1 °C. The water maze was divided into four equal quadrants, with the second quadrant featuring a circular platform with a diameter of 10 cm fixed 2 cm below the water’s surface. Rats were given visual signals inside the pool to memorize, while the area outside the pool was covered in drapes to restrict their visibility. The hidden platform test lasted five days (twice a day). During training, rats were gently placed into the water while facing the wall of the pool, with the quadrant of entry changing for each session. When rats were placed in the pool, a tracking system was utilized to record their route and timing. If rats could find the platform, the timer was stopped and they were allowed to stay on it for 3–6 sec; if rats could not discover the platform within 60 sec, they were instructed to stay on it for 3–6 sec. The time taken by rats to reach the platform each time was recorded as the latency to escape. On the 6th day, the platform was removed and the probe trial was started by placing rats in a pool of water and recording the speed, total distance, number of times they crossed the platform, and time spent in the quadrant where the platform was located in 60 sec.

### 2.3 Animal dissection and sampling

The animals were anaesthetized and dissected at 8 a.m. the day after the noise exposure and behavioral experiments were completed. The anaesthesia was induced in the rats by the intraperitoneal injection of 3% sodium pentobarbital at a concentration of 0.18 mL/100 g. The depth of anaesthesia was determined by stimulating the toes or paw area of the rats. If there was no response, the anaesthesia was considered to be adequate. Once the depth of anaesthesia had been confirmed, the rats were dissected and samples were taken.

### 2.4 Western blot

Hippocampal tissues were lysed using lysis solution (PC201; Epizyme, Hangzhou, China), and the supernatant was collected for protein level analysis following sufficient grinding and 10-min centrifugation at 12,000 rpm (4 °C). The primary antibodies used in this study were summarized as follows: mouse Glutathione Peroxidase 4 (sc-166570, 1:1000; Santa Crutz Biotechnology, San Diego, CA, USA), mouse ferritin heavy chain (sc-376594, 1:1000; Santa Crutz Biotechnology), mouse GFAP (sc-166458, 1:1000; Santa Crutz Biotechnology), mouse Iba1 (sc-32725, 1:1000; Santa Crutz Biotechnology), rabbit xCT/SLC7A11 (12691, 1:1000; Cell Signaling Technology, Danvers, MA, USA), rabbit APP/Beta Amyloid Polyclonal antibody (25524-1-AP, 1:1000, Proteintech, Rosemont, IL, USA), rabbit Tau-S396 (AP1028, 1:1000; ABclonal, Woburn, MA, USA), mouse anti-Actin (GB15001-100, 1:1000; Servicebio, Wuhan, China), and mouse anti-Tubulin (GB122667-100, 1:1000; Servicebio). The secondary antibodies used were anti-mouse IgG and HRP-linked antibodies (GB23301, 1:3000; Servicebio) and anti-rabbit IgG and HRP-linked antibodies (GB23303, 1:3000; Servicebio). Detection was performed by a chemiluminescence kit (SQ201, Epizyme), followed by quantitative analysis using a chemiluminescence immunoassay analyzer (5200Multi, Tanon, China) and ImageJ software.

### 2.5 Analysis of oxidative stress levels

Malondialdehyde (MDA) kit (G4300, Servicebio), Total Superoxide Dismutase (SOD) kit (A001-1; Njjcbio, Beijing, China), Glutathione (GSH) kit (G4305, Servicebio), and Glutathione Peroxidase (GSH-Px) kit (A005, Njjcbio) were utilized to measure oxidative stress level. All measurements were performed according to the manufacturers’ instructions.

### 2.6 HE staining

The brains of rats were sectioned into paraffin slices, which were then dewaxed and stained with hematoxylin, dehydrated with alcohol, and subsequently stained with eosin (G1003, Servicebio). They were thereafter dehydrated and sealed, and the cell morphology was observed using a microscope.

### 2.7 Immunohistochemical

Paraffin sections of rat brain were deparaffinized with primary antibodies mouse GFAP (sc-166458, 1:500; Santa Crutz Biotechnology), rabbit Iba-1 (GB153502-100, 1:500; Servicebio) and rabbit GPX4 (TA376761, 1:100; ORIGENE) were incubated overnight at 4 °C. The secondary antibodies used were anti-mouse IgG and HRP-linked antibodies (GB23301, 1:500; Servicebio) and anti-rabbit IgG and HRP-linked antibodies (GB23303, 1:500; Servicebio). They were thereafter dehydrated and sealed, and the cell morphology was observed using a microscope.

### 2.8 RNA-seq

Total RNA was extracted from 6 hippocampal tissues (CON group (3) vs. NE group (3)) using Trizol reagent (G3013, Servicebio), and reversely transcribed it into cDNA utilizing a reverse-transcription kit. The RNA samples were tested for eligibility, and the samples were thereafter subjected to sequencing library construction.

### 2.9 Differential expression analysis

The data from the two groups were analyzed for differential expression using the DESeq2 R package (1.20.0) with *P* < 0.05 set to obtain differentially expressed genes (DEGs). The data were visualized using the pheatmap package (1.0.12), the ggplots package (3.4.4), the limma package (3.58.1), and the VennDiagram package (1.7.3). Notably, 1456 oxidative stress-related genes were attained from the Genecard database (https://www.genecards.org/). Moreover, 1256 ferroptosis-related genes were acquired from the Ferroptosis database (http://www.zhounan.org/ferrdb) and the Genecard database.

### 2.10 Enrichment analyses

Visualization was carried out using the clusterProfiler package (4.10.0). The DEGs were individually analyzed using the Gene Ontology (GO), Kyoto Encyclopedia of Genes and Genomes (KEGG), and Gene Set Enrichment Analysis (GSEA) enrichment analyses. Statistical significance was defined as *P* < 0.05. Enrichment analysis of DEGs was undertaken utilizing Metascape (https://metascape.org). The results were visualized using the ggplots package (3.4.4).

### 2.11 Protein-protein interaction (PPI) network analysis and the hub genes

PPI networks and hub gene networks were constructed using STRING (https://cn.string-db.org/), as well as Cytoscape (version 3.9.1) and its associated plugins. The upstream miRNAs of hub genes were predicted using Cytoscape (3.9.1) and its associated plugins.

### 2.12 Diagnostic receiver operating characteristic (ROC) curve

GSE132903 was downloaded from the Gene Expression Omnibus (GEO) database (https://www.ncbi.nlm.nih.gov/geo/). Validating the predictive ability of DEGs based on 98 controls and 97 AD patients. The results were visualized using the pROC package (1.18.0).

### 2.13 MR analysis

Exposure and outcome data were retrieved from IEU openGWAS (https://gwas.mrcieu.ac.uk). The dataset, attained from the European population, encompassed 37,154 controls and 17,008 AD patients, and included protein level of Rarres2. The MR analysis and visualization were undertaken by the TwoSampleMR package (0.5.8). MR analysis uses genetic variation as an instrumental variable (IV) to estimate the causal relationship between exposure and outcome. The significance threshold was set to *P* < 5 × 10^−6^ to screen for SNPs with strong correlation with exposure. Moreover, The parameters r^2^ equal to 0.001 and kb equal to 10,000 were subsequently implemented to filter out SNPs exhibiting linkage disequilibrium, and phenoscanner (http://www.phenoscanner.medschl.cam.ac.uk/) was employed to exclude confounding factors [19]. Cochran’s Q test was utilized to evaluate heterogeneity among IVs, with a P-value greater than 0.05 indicating no significant heterogeneity. Horizontal pleiotropy was identified if the inverse variance weighted regression intercept deviated significantly from 0 with a P-value less than 0.05. Finally, reverse MR analysis was conducted to verify the absence of reverse causality between exposure and outcome, with a P-value greater than 0.05 indicating no such reverse causality [22].

### 2.14 Statistical analysis

All data were expressed as mean ± standard error of the mean (SEM) and were analyzed using SPSS 25.0 software (IBM, Armonk, NY, USA). The two groups were statistically compared using the two-sample independent t-test. If normality and Chi-square criteria would be met, differences between the two groups were analyzed using one-way analysis of variance (ANOVA). Otherwise, nonparametric tests were performed. *P* < 0.05 was considered statistically significant. All experiments were repeated at least 3 times.

## 3. Results

### 3.1 Chronic noise exposure causes cognitive impairment in rats

**Fig. 1:**
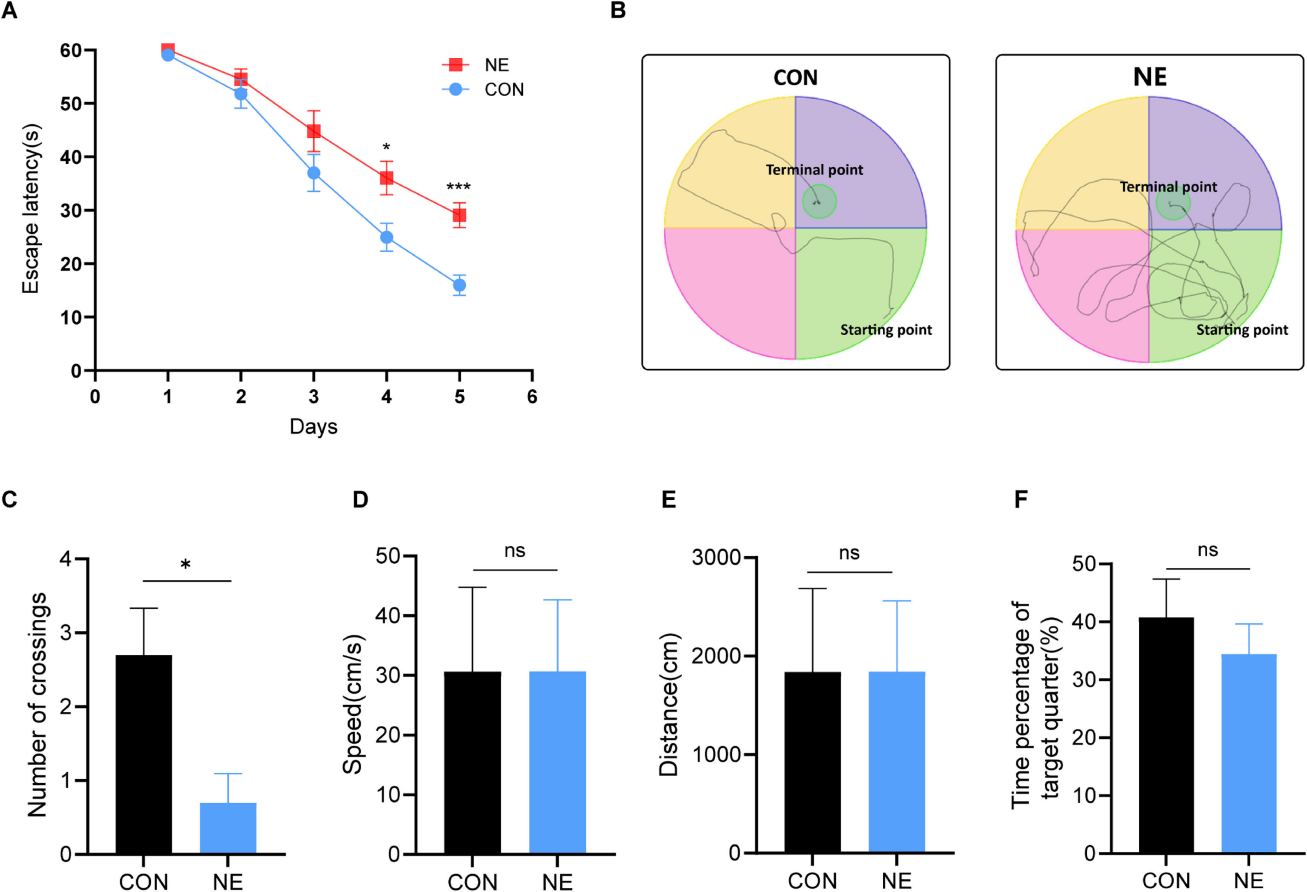
The effects of noise on learning and cognitive functions of rats. (A) The escape latency of rats. (B) The movement trajectory of rats in MWM. (C) The number of crossing the platform. (D–F) The swimming speed (D), swimming distance (E), and percentage of stay time in quadrant of platform (F) of rats. *, *P* < 0.05, ***, *P* < 0.001.

The effects of chronic noise exposure on the cognitive function of rats were assessed using the MWM test. In the hidden platform task, rats in the NE group had significantly longer escape latency than those in the CON group from day 4 to day 5 (Fig. 1A). As illustrated in Fig. 1B, rats in the NE group traveled significantly more distance to reach the platform than those in the CON group. In the exploratory test (day 6), the number of times crossed the platform within a certain period in the exposed group was significantly lower than that in the CON group, while there were no significant differences in swimming speed, total distance traveled, and activity time in the quadrant where the platform was located between the two groups (Fig. 1C–F). The results indicated that the noise exposure impaired the cognitive function of rats. However, there was no significant difference in the exercise level of rats between the two groups.

### 3.2 Chronic noise exposure causes AD-like neuropathology and neuronal damage in rats

**Fig. 2:**
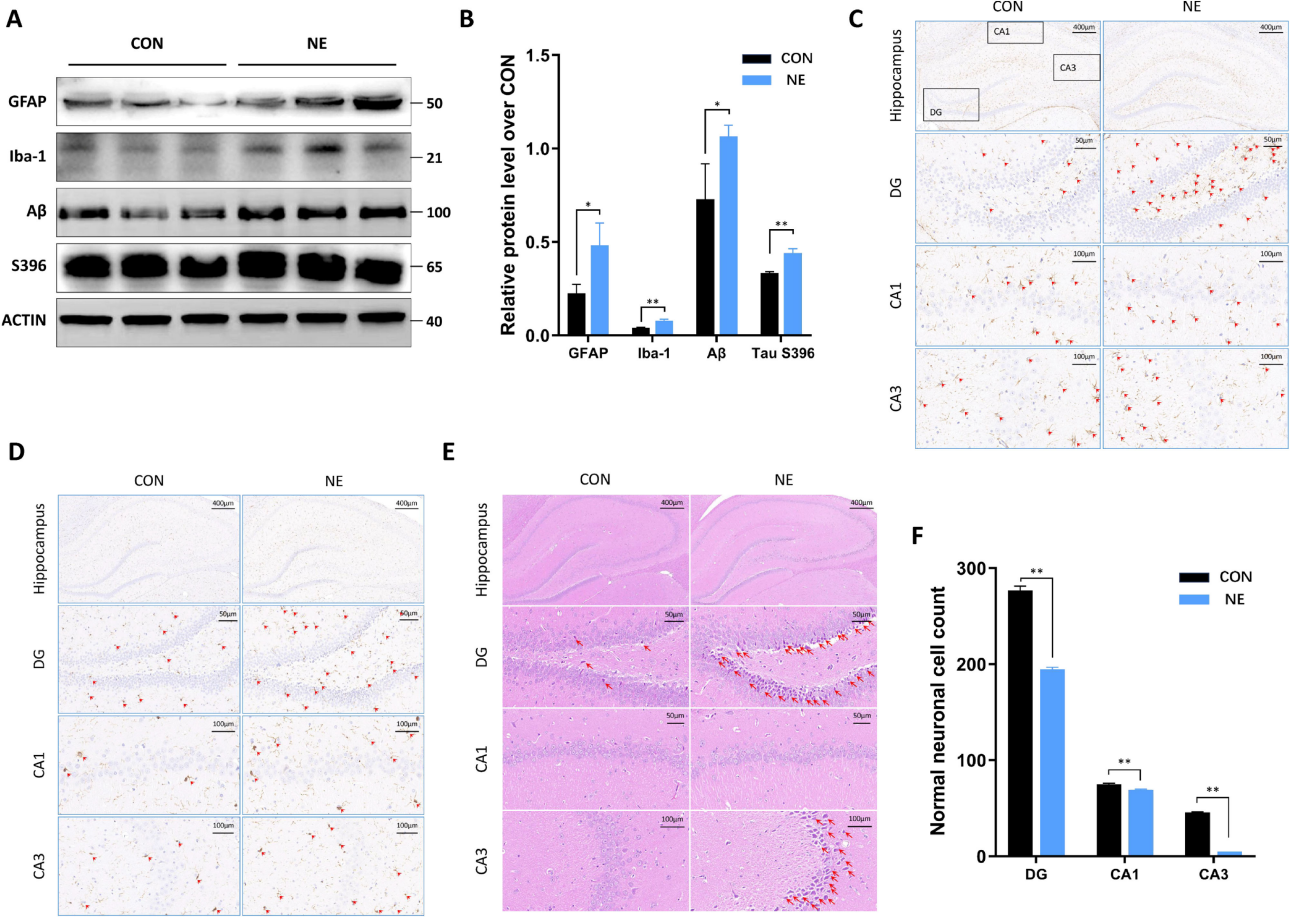
Noise exposure induced AD-like neuropathology and neuronal damage. (A–B) Changes in expression levels of GFAP, Iba-1, Aβ, and S396 in the rat hippocampus (n = 3). (C) Immunohistochemistry of GFAP in rat hippocampus. Red arrows represent GFAP-positive areas. (D) Immunohistochemistry of Iba-1 in rat hippocampus. Red arrows represent Iba-1-positive areas. (E) HE staining of rat hippocampal sections. Red arrows represent damaged neuronal cells. (F) Normal neuronal cell count in rat hippocampus. *, *P* < 0.05, **, *P* < 0.01

To investigate the effects of chronic noise exposure on AD-like neuropathology and neuronal structure in rat hippocampus, the expression levels of glial fibrillary acidic protein (GFAP), Ionized calcium-binding adapter molecule 1 (Iba-1), Aβ, and Tau S396 were determined in rat hippocampal tissues, and neuronal changes in hippocampal tissues were assessed using HE staining. As displayed in Fig. 2A–B, the protein expression levels of GFAP, Iba-1, Aβ, and Tau S396 were significantly higher in the NE group than those in the CON group. Furthermore, immunohistochemistry was employed to assess the expression of GFAP and Iba-1 in the hippocampus. As illustrated in Fig. 2C and D, the levels of GFAP and Iba-1 were significantly elevated in both the CA3 region and the DG region in the NE group relative to the CON group. This is evidenced by the fact that the cell protrusions indicated by the red arrows became more prominent and densely packed. These findings indicate that chronic noise exposure may induce AD-like neuropathology.

In addition, the HE staining results indicated that neurons in the CON group were neatly arranged without obvious damage, while the number of neurons in the NE group was significantly reduced, with wrinkled cells and deeply stained nuclei (Fig. 2E and F). The locations to be noted in the HE-stained sections of the two groups were marked with red arrows, as shown in Fig. 2E. The cells indicated by these arrows exhibited wrinkled morphology and deeply stained nuclei, which indicated that the neurons had been damaged [23]. In order to ascertain the number of normal neurons in the hippocampus of the two groups, as shown in Fig. 2F, histograms were employed to enumerate the normal neuronal cells within the sectioned field of view. The results demonstrated that the number of normal neuronal cells in the CON group was significantly higher than that in the NE group in the CA3 and DG regions. These findings indicate that chronic noise exposure may result in neuronal death in the rat hippocampus.

### 3.3 Chronic noise exposure induces ferroptosis in the hippocampus of rats

**Fig. 3:**
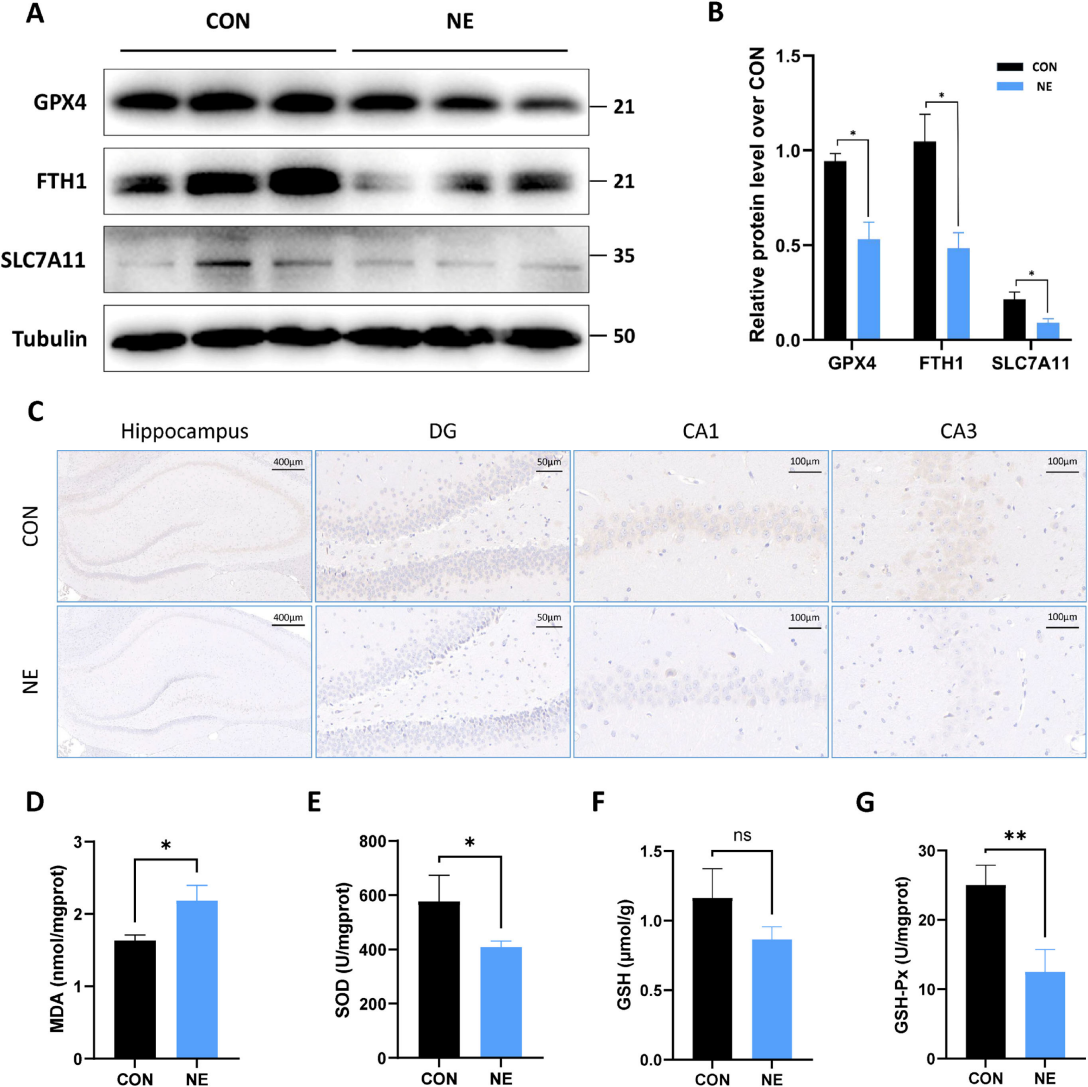
Noise exposure induced ferroptosis in hippocampus of rats. (A–B) Changes in expression levels of GPX4, FTH1, and SLC7A11 in the rat hippocampus (n = 3). (C) Immunohistochemistry of GPX4 in rat hippocampus. (D–G) Changes in levels of MDA, SOD, GSH, and GSH-Px in the rat hippocampus. *, *P* < 0.05, **, *P* < 0.01.

To explore whether ferroptosis occurs in the rat hippocampus after chronic noise exposure, the expression levels of GPX4, FTH1, and SLC7A11 in the hippocampus of two groups of rats were examined and quantitatively analyzed. The results indicated that the expression levels of GPX4, FTH1, and SLC7A11 in the hippocampus of rats in the NE group were significantly lower than those in the CON group (Fig. 3A–B). In addition, we examined the expression level of GPX4 in the hippocampus of both groups of rats by immunohistochemistry. As shown in Fig. 3C, the GPX4 expression level in the NE group was significantly lower than that in the CON group, which is consistent with our protein level results.

In addition, noise exposure increased the MDA level (product of LPO) in the hippocampus (Fig. 3D), and the levels of SOD and GSH-Px were significantly reduced, while there was no significant difference in the GSH level (Fig. 3E–G). These results suggested that chronic noise exposure could induce ferroptosis in the hippocampus of rats.

### 3.4 Identification of DEGs in chronic noise-exposed rats

**Fig. 4:**
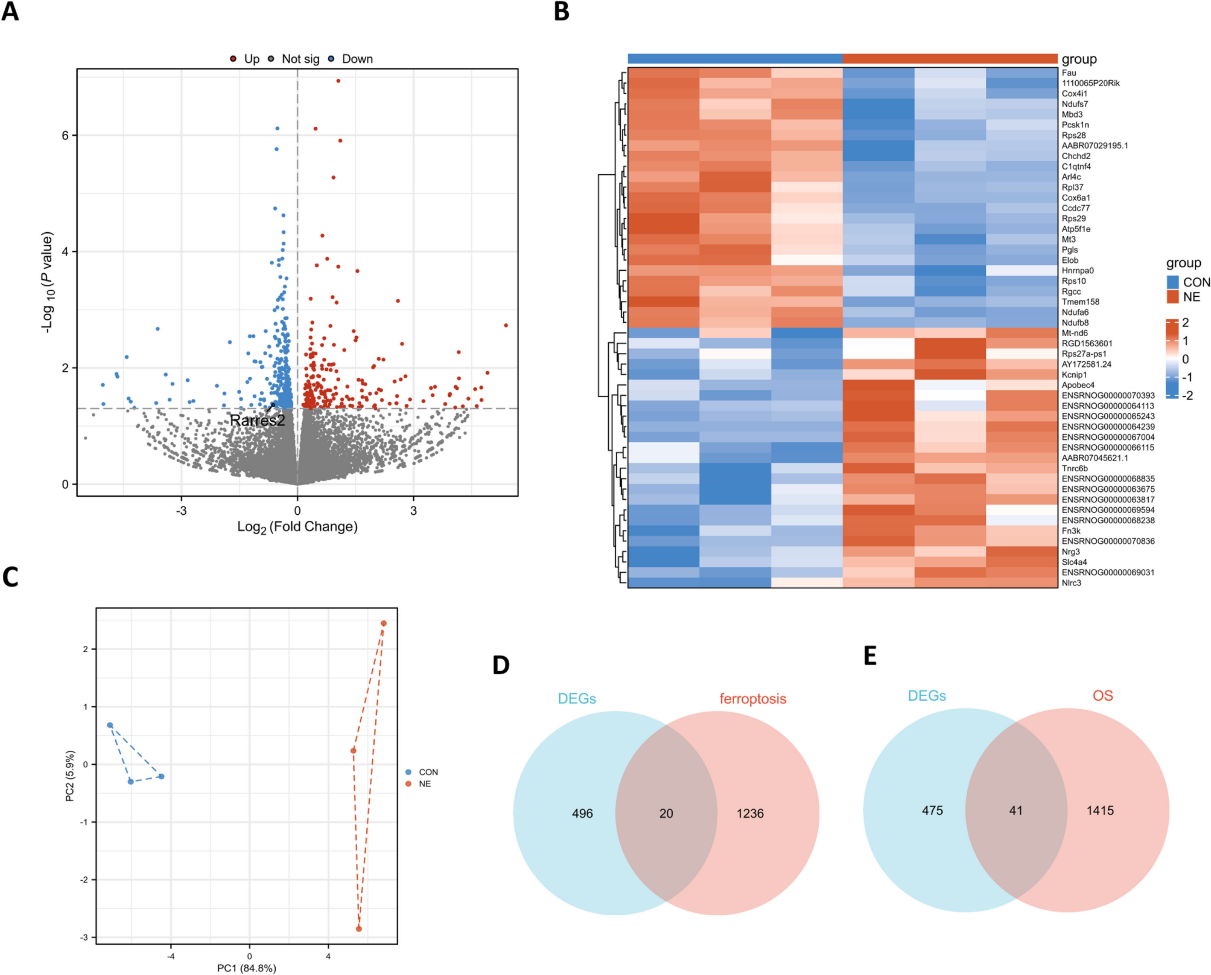
Identification of DEGs in noise-exposed rats. (A) The volcanic map of DEGs. (B) The heatmap displaying the top 25 upregulated DEGs and top 25 downregulated DEGs. (C) The gene cluster in PCA loading score. (D) The Veen plot of DEGs and ferroptosis-related genes. (E) The Veen plot of DEGs and oxidative stress-related genes.

Totally, 516 DEGs were obtained using the limma package, including 217 upregulated and 299 down-regulated DEGs (Fig. 4A–C). As displayed in Fig. 4A, Rarres2 level in the NE group was significantly lower than in the CON group. Using Veen plots for ferroptosis-related genes and oxidative stress-related genes to intersect with DEGs, 20 ferroptosis-related DEGs and 41 oxidative stress-related DEGs were obtained (Fig. 4D–E). Besides, the genes with |Log2 (fold-change)| > 0.585 in ferroptosis-related DEGs were *Rarres2*, *Klf2*, *Tfap2c*, and *Angptl7*; the genes with |Log2 (fold-change)| > 0.585 in oxidative stress-related DEGs were *Slc4a1*, *Esr1*, *Acox2*, *Endog*, *Cyba*, *Klf2*, *Ddc*, and *Tp73*.

### 3.5 Functional enrichment analysis of DEGs

**Fig. 5:**
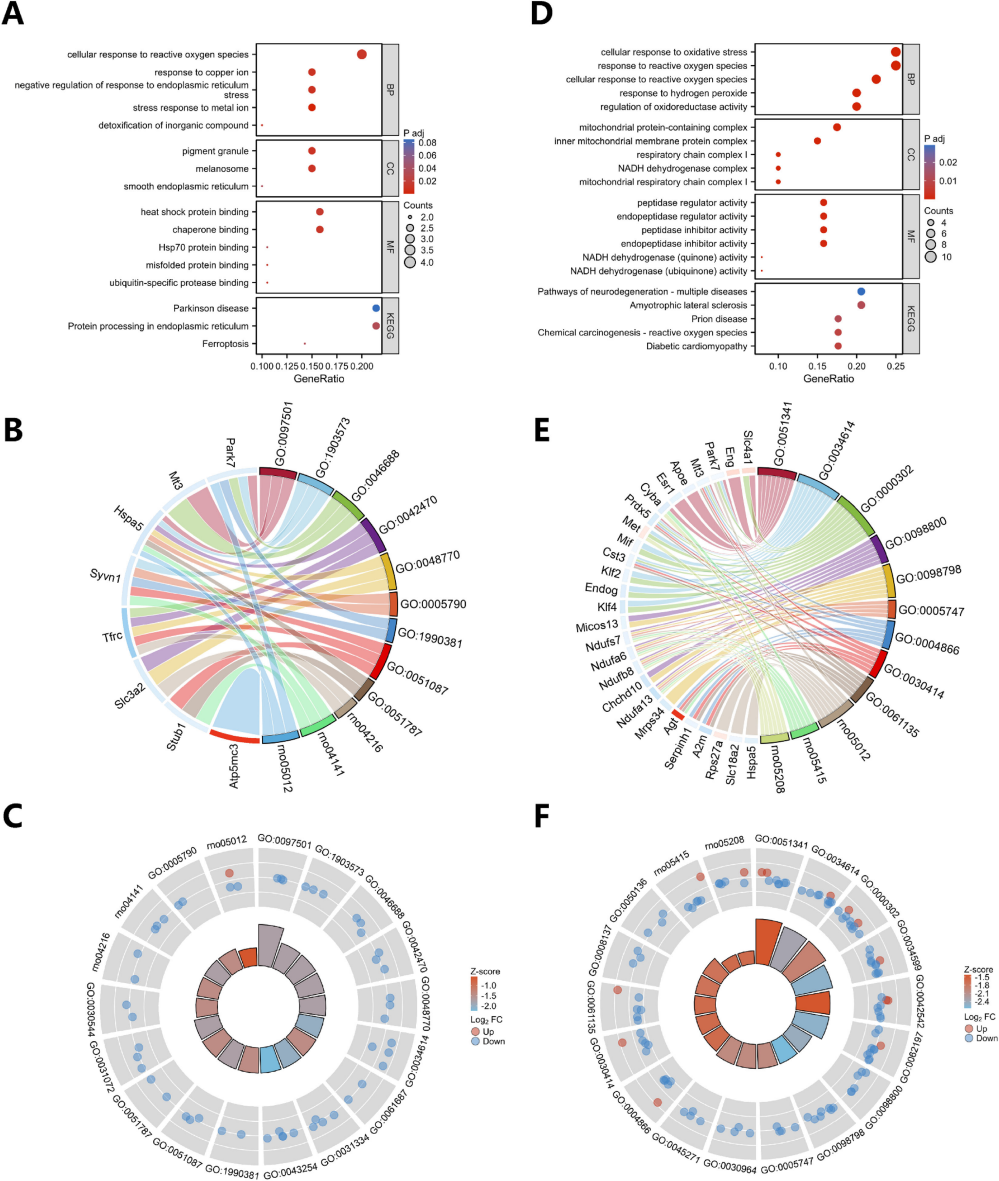
Functional enrichment analysis of oxidative stress-related DEGs and ferroptosis-related DEGs. (A) The enriched GO terms of oxidative stress-related DEGs. (B) The enriched GO terms of ferroptosis-related DEGs. (C) The enriched KEGG pathways of oxidative stress-related DEGs. (D) The enriched KEGG pathways of ferroptosis-related DEGs.

The results of the functional enrichment analysis of ferroptosis-related DEGs and oxidative stress-related DEGs are illustrated in Fig. 5. Figure 5A–C displays the bar chart of the GO and KEGG pathway enrichment analyses of ferroptosis-associated DEGs, involving the stress response to metal ion, the negative regulation of response to endoplasmic reticulum stress, the cellular response to reactive oxygen species, the response to copper ion, ferroptosis, protein processing in endoplasmic reticulum, and Parkinson’s disease.

Figure 5D–F depicts the results of the GO and KEGG pathway enrichment analyses of oxidative stress-related DEGs, involving the regulation of oxidoreductase activity, the cellular response to reactive oxygen species, the response to reactive oxygen species, the cellular response to reactive oxygen species, the response to hydrogen peroxide, diabetic cardiomyopathy, chemical carcinogenesis-reactive oxygen species, and pathways of neurodegeneration-multiple diseases.

### 3.6 PPI network analysis

**Fig. 6:**
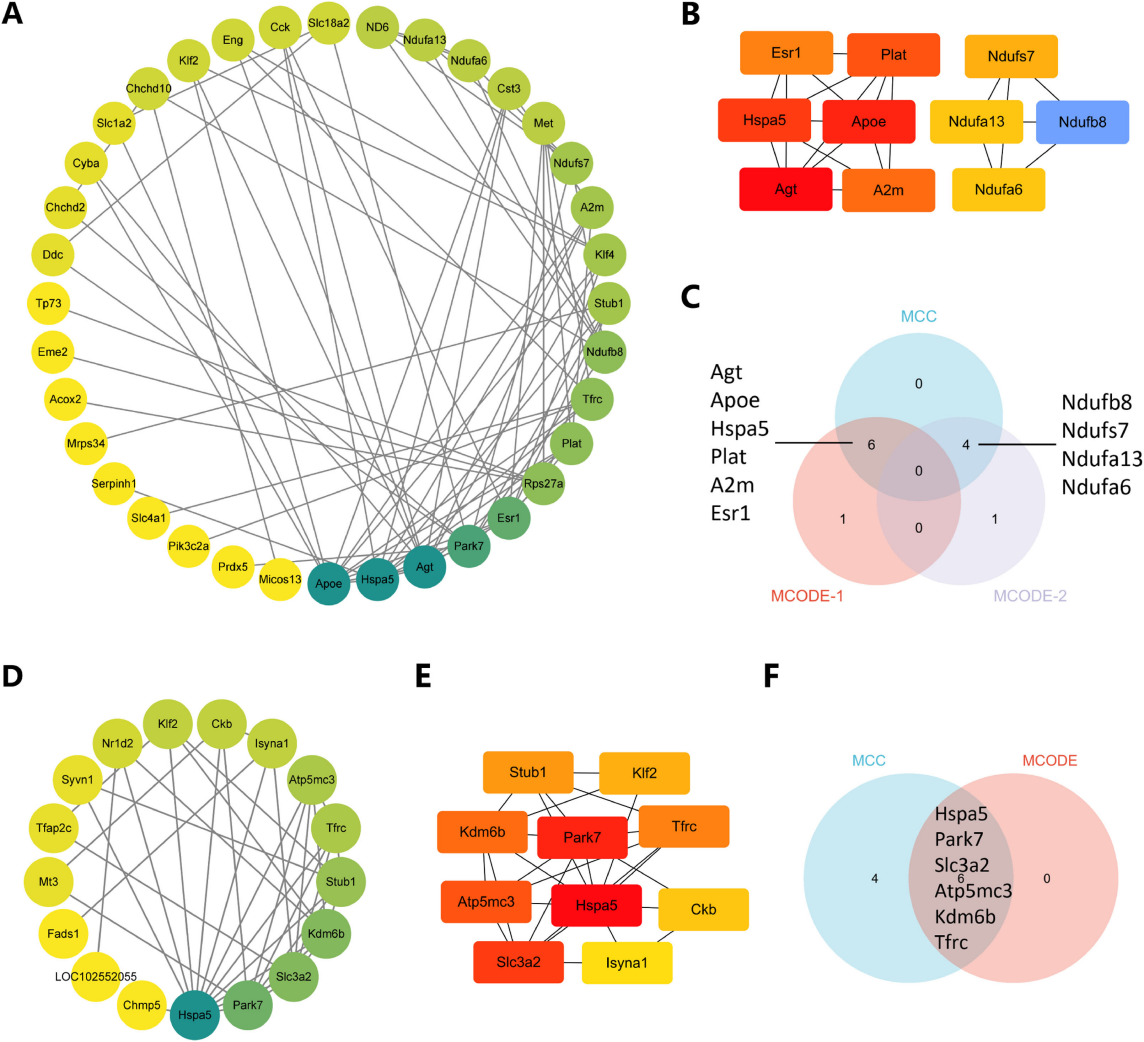
The PPI networks of oxidative stress-related DEGs and ferroptosis-related DEGs. (A) The PPI network among oxidative stress-related DEGs illustrates darker colors, indicating higher interconnectivity among molecules. (B) The top 10 ranked oxidative stress-related DEGs identified using MCC. (C) The Veen plots of genes obtained from MCC and MCODE. (D) In the PPI network among ferroptosis-related DEGs, darker shades indicate increased molecular connections. (E) The top 10 ranked ferroptosis-related genes identified using MCC. (F) The Veen plots of genes obtained from MCC and MCODE.

The PPI networks of oxidative stress-associated DEGs and ferroptosis-associated DEGs are illustrated in Fig. 6. Figure 6A shows the PPI network between oxidative stress-related DEGs, and the top 10 ranked molecules were selected to further construct the PPI network according to the ranking of the Cytoscape plug-in MCC (Fig. 6B). Subsequently, the oxidative stress-related DEGs were clustered using the Cytoscape plug-in MCODE, and the clustered molecules were intersected with the top 10 molecules ranked by the MCC to achieve 10 hub genes (Fig. 6C). Using the same method to analyze ferroptosis-related DEGs, 2 PPI networks and 6 hub genes were obtained (Fig. 6D–F).

The oxidative stress-related hub genes were combined with ferroptosis-related hub genes, and functional enrichment analysis was conducted on the 12 genes obtained (Supplementary Fig. 2). Supplementary Fig. 2A displays the results of the GO and KEGG pathway enrichment analyses for these 12 genes, and Supplementary Fig. 2B depicts the network of the enriched pathways. In addition, Metascape was utilized for enrichment analysis of these genes, and the networks of enrichment pathways were constructed (Supplementary Fig. 2C–D).

### 3.7 Validation of genes using ROC curve analysis

**Fig. 7:**
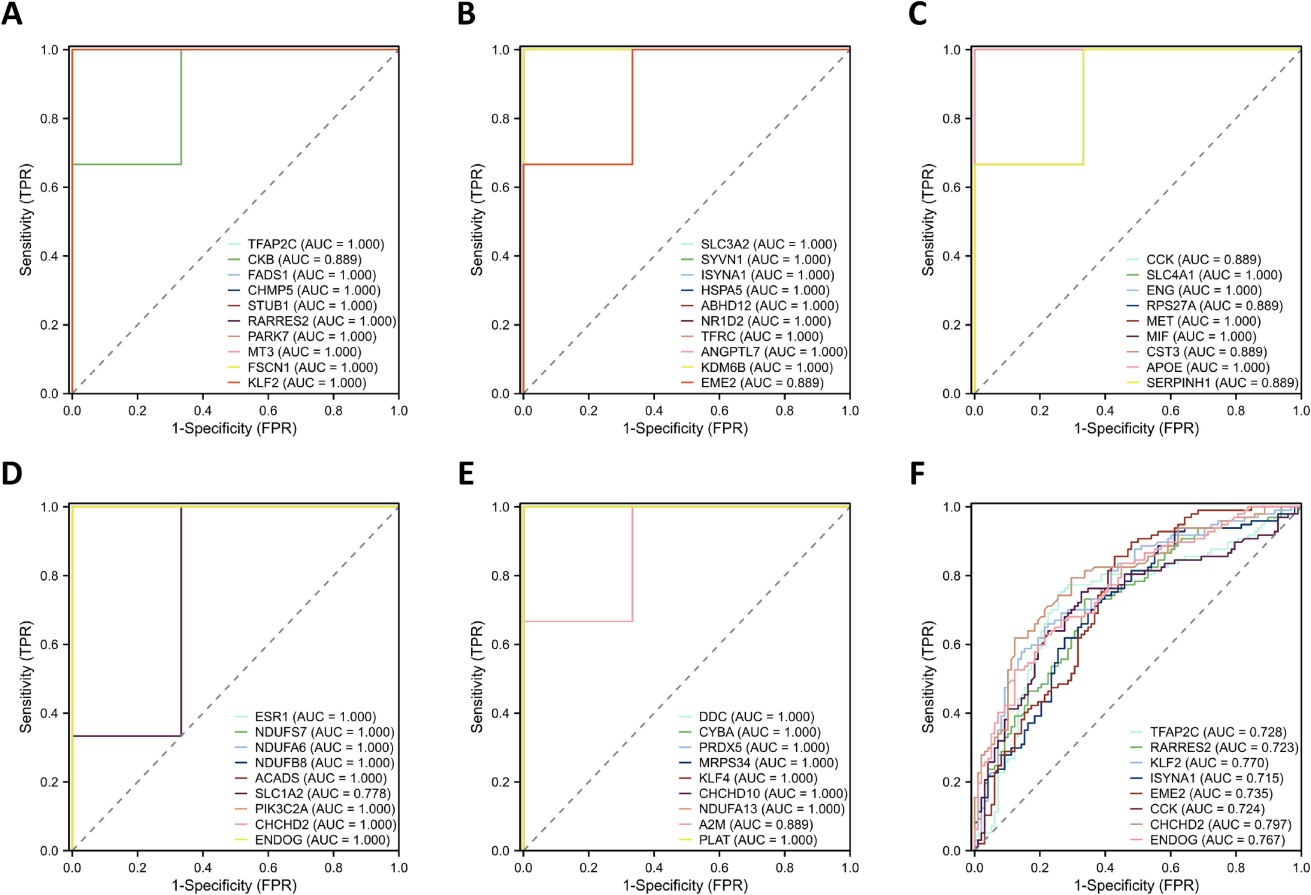
Validation of genes using ROC curves. (A–E) ROC curves for oxidative stress-related DEGs and ferroptosis-related DEGs in NE. (F) ROC curves for oxidative stress-related DEGs and ferroptosis-related DEGs in AD with AUC > 0.7.

Given the AD-like neuropathology in the rat hippocampus following noise exposure, AD-related datasets were acquired from the GEO to assess the predictive ability of DEGs. The ROC curve analysis was employed to explore the predictive ability of these genes in distinguishing between the CON group and noise group, as well as between controls and AD patients. An area under the curve (AUC) greater than 0.7 was indicative of a notable predictive ability (Fig. 7A–F).

### 3.8 MR analysis of Rarres2 on AD

**Fig. 8:**
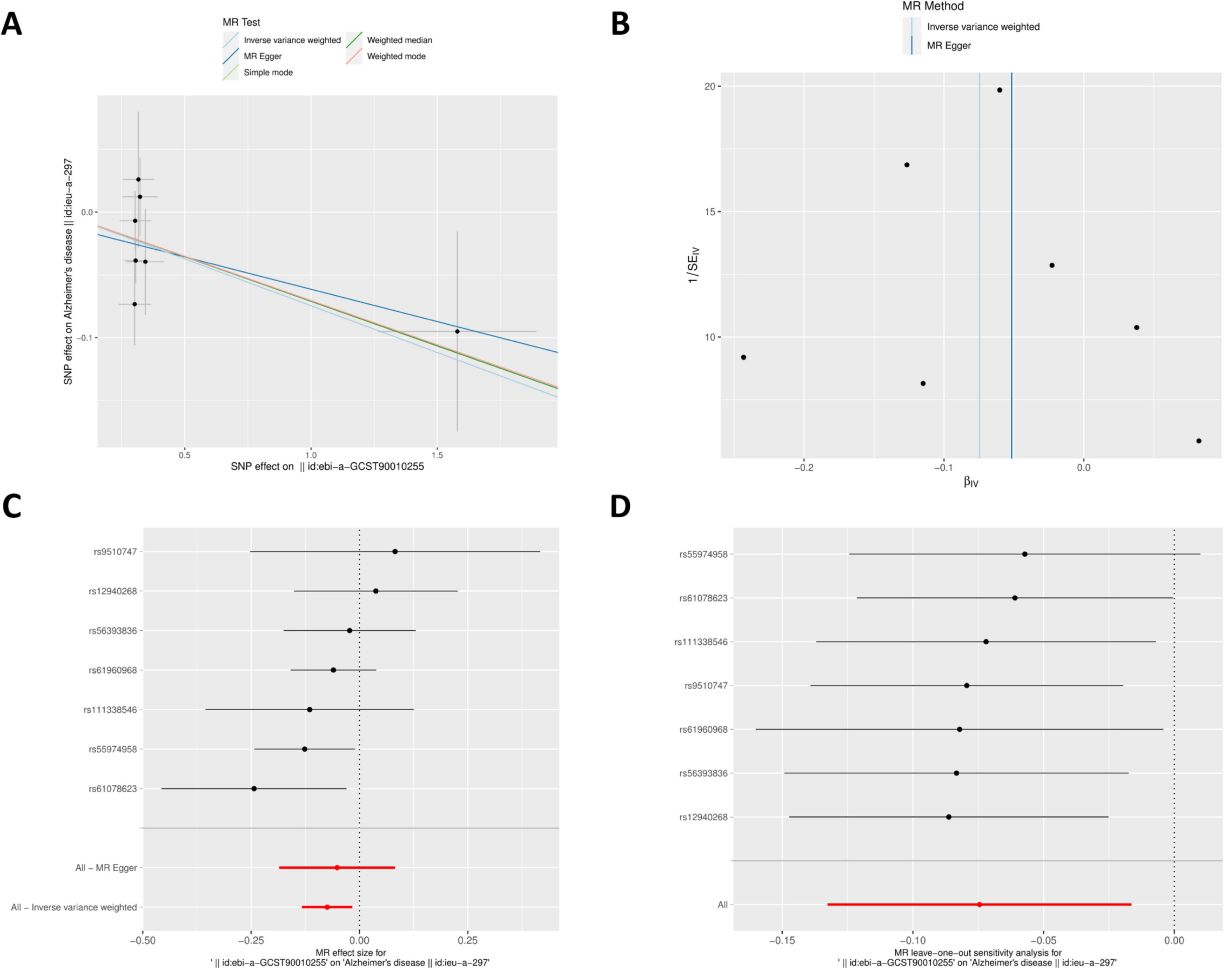
Visualization of the MR analysis of Rarres2 on AD. (A) The SNP effect sizes for Rarres2 and AD. (B) The funnel plot displaying no significant heterogeneity among IVs of Rarres2. (C) The forest plots of causal effects of Rarres2 SNPs on AD. (D) The leave-one-out analysis of the effect of Rarres2 on AD.

According to the AD-like neuropathology of the rat hippocampus following noise exposure and the lack of cognitive impairment-related datasets in the database, AD-related data were analyzed. Supplementary Fig. 3 visualizes the results of MR analysis of the relationship between Rarres2 and AD. In the bidirectional MR analysis, 7 SNPs that were strongly correlated with Rarres2 were obtained as the IVs of Rarres2 and AD as the outcome. The results of MR analysis of the correlation between Rarres2 and AD revealed that the inverse variance weighted (IVW) method (odds ratio (OR) = 0.928, 95% confidence interval (CI) = 0.876–0.984, *P* = 0.012), MR-Egger’s method (OR = 0.950, 95%CI = 0.831–1.086, *P* = 0.485), and weighted median method (OR = 0.931, 95%CI = 0.862–1.006, *P* = 0.072) had significant results. These results indicated that individuals with Rarres2 had a 0.928 times higher risk of being genetically predisposed to AD compared to those without Rarres2 (Supplementary Table 1 and Fig. 8). The Cochran’s Q analysis exhibited no heterogeneity among IVs (Supplementary Table 2, *P* > 0.05).

The scatterplot in Fig. 8A displays the SNP effect sizes for Rarres2 and AD. The funnel plot in Fig. 8B illustrates that the IVs of Rarres2 are symmetric in the IVW, and the MR-Egger’s test exhibited no horizontal pleiotropy in these IVs (Supplementary Table 3, Fig. 8C). The Leave-one-out sensitivity analysis revealed that removing each SNP one by one did not remarkably impact the results, and almost all lines were on the right side of 0 (Fig. 8D), indicating that the results of MR analysis were relatively reliable. Collectively, these results suggested a negative genetic relationship between Rarres2 and AD.

### 3.9 Reverse MR analysis of AD on Rarres2

In the reverse MR analysis, AD and Rarres2 were taken as the exposure and outcome into account respectively, to obtain 32 SNPs that were strongly associated with AD as IVs. The reverse MR analysis results are illustrated in Supplementary Table 1 and Supplementary Fig. 5. It was revealed that there was no causal relationship between AD and Rarres2. Cochran’s Q analysis indicated that there was no heterogeneity among the IVs (Supplementary Table 2, *P* > 0.05). The MR Egger’s test indicated the absence of horizontal pleiotropy among these IVs (Supplementary Table 3). The results of leave-one-out analysis demonstrated no abnormality in the IVs. Taken together, these results did not support an inverse genetic relationship between Rarres2 and AD.

## 4. Discussion

It is a stereotype that noise causes hearing loss, and studies have demonstrated that the hippocampus is more susceptible to noise stimulation than the auditory cortex, and this hippocampal damage mainly goes unnoticed by several individuals [24–26]. Cui et al. pointed out that noise could induce inflammation and disruption of autophagic flow in the hippocampus, while the involvement of ferroptosis has been rarely investigated [11, 27]. The present study, for the first time, assessed the involvement of ferroptosis in chronic noise-induced cognitive impairment and AD-like neuropathology.

Previous studies have reported that noise could cause cognitive impairment and AD-like neuropathology [27, 28]. In the present study, Wistar rats were used for modeling, and the results of the MWM experiment confirmed that noise exposure impaired learning and memory functions in rats, indicating that the model of chronic noise-induced cognitive impairment was successfully established. Aβ, a polypeptide resulting from the degradation of amyloid precursor protein (APP) by β- and γ-secretases, exerts neurotoxic effects [29]. Tau protein, primarily expressed in the brain as a microtubule-associated protein, plays a role in stabilizing axonal microtubules. However, its stabilizing effect diminishes when Tau protein undergoes hyperphosphorylation [30]. The present study revealed that chronic noise exposure resulted in the development of AD-like neuropathology by causing Aβ buildup, hyperphosphorylation of Tau in the hippocampus, and neuronal injury as evidenced by HE staining and Western blotting results [23]. Furthermore, the expression levels of the microglia and astrocyte markers, Iba-1 and GFAP, were elevated, indicating that noise could activate these cells and potentially cause neuroinflammation [31].

The Western Blot analysis in this study reveals that chronic noise exposure is linked to elevated protein levels of GFAP, Iba-1, Aβ, and Tau S369 in the hippocampus, suggesting that such exposure may induce Alzheimer’s disease-like pathological changes. To pinpoint the target cells affected by noise exposure in the hippocampus, immunohistochemistry was used to examine GFAP and Iba-1 expression in various hippocampal regions. Results showed significantly higher expression levels of GFAP and Iba-1 in the DG and CA3 regions of the noise exposure (NE) group compared to the control group. Additionally, HE staining indicated more severe neuronal damage in the DG and CA3 regions of the NE group, suggesting these subregions are particularly vulnerable to chronic noise exposure. Therefore, future research should focus on the target cells in these two regions.

Neurons, as the longest surviving terminally differentiated cells in the body, are subjected to several cellular stresses during aging, of which oxidative stress is noteworthy [32, 33]. Due to its high oxygen consumption, abundance of redox-active metals, and presence of polyunsaturated fatty acids prone to damage by ROS, alongside its relatively modest antioxidant defenses, it exhibits an elevated susceptibility to oxidative stress. Intracellular iron may also be involved in oxidative stress, which can worsen the condition and cause cell death, and this phenomenon is referred to as ferroptosis [34, 35]. Ferroptosis is involved in several neurodegenerative diseases, and it has been demonstrated that inhibiting ferroptosis can alleviate AD-like neuropathology and cognitive deficits in the AD model [14, 36, 37]. The present study aimed to investigate whether ferroptosis could be involved in chronic noise-induced cognitive impairment.

Ferroptosis is mainly induced by iron metabolism, lipid metabolism, amino acid metabolism, and other processes [38]. The glutathione metabolic pathway plays a notable role in the regulation of ferroptosis. GSH is an important intracellular antioxidant synthesized from glutamate, cysteine, and glycine, and its level is negatively correlated with ferroptosis. Glutathione peroxidase 4 (GPX4) relies on GSH as both a cofactor and synthetic substrate to execute its lipid repair function [39]. The presence of GSH is essential for GPX4 activity. Specifically, intracellular lipid peroxidation increases when GSH level diminishes, leading to GPX4 inactivation and triggering ferroptosis [40]. SLC3A2 and SLC7A11 dimers, positioned on the cell membrane’s surface, constitute System Xc. SLC7A11 is the primary component responsible for translocating cystine into the cytosol for GSH production. Therefore, ferroptosis develops when SLC7A11 expression level is downregulated [41]. Ferritin consists of two types of subunits: the ferritin heavy chain (FTH1), which has ferrous oxidase activity and converts Fe (II) to Fe (III) for safe storage, and the ferritin light chain (FTL) [14]. Ferroptosis can be caused by ferritin degradation or downregulation of FTH1 expression level [12, 42, 43]. The results of the present study indicated that noise exposure resulted in the downregulation of the expression levels of GPX4, SLC7A11, and FTH1, while a decrease in the levels of SOD and GSH-Px and an increase in the level of the lipid peroxidation product MDA were found. These findings indicated that chronic noise exposure could induce ferroptosis in rat hippocampus [44].

The present research assessed the role of ferroptosis in chronic noise-induced cognitive impairment by multi-perspective analysis and bioinformatics visualization of the relevant DEGs. After combining data from the Genecard database with sequencing data, 41 oxidative stress-related DEGs and 20 ferroptosis-related DEGs were identified. These DEGs thereafter underwent the GO and KEGG pathway enrichment analyses, PPI network analysis, and ROC curve analysis.

The results of the GO enrichment analysis included three GO components, such as biological process (BP), cell component (CC), and molecular function (MF). The BP enrichment analysis revealed that ferroptosis-related DEGs were associated with various functions, including stress response to metal ions, negative regulation of response to endoplasmic reticulum stress, response to copper ions, cellular response to reactive oxygen species, and detoxification of inorganic compounds. Notably, these genes were all downregulated after noise exposure, suggesting a negative regulation of these BPs that emerged protective against stress and inorganic toxicity, such as iron following noise exposure. Meanwhile, the results of CC enrichment indicated that the expression levels of ferroptosis-related DEGs involved in endoplasmic reticulum components were also downregulated following noise exposure, possibly suggesting the role of the endoplasmic reticulum in noise-induced ferroptosis [45]. The results of MF enrichment revealed that the ferroptosis-related DEGs were involved in the ubiquitin-specific protease binding, chaperone binding, misfolded protein binding, heat shock protein binding, and Hsp70 protein binding. The results of the KEGG pathway enrichment analysis indicated that the ferroptosis-related DEGs were found to be associated with protein processing in the endoplasmic reticulum and Parkinson’s disease pathways, including Hspa5. Hspa5 is a heat shock protein, playing a variety of important cellular functions. It has been demonstrated that Hspa5 can inhibit P53 expression level, leading to high expression levels of GPX4 and SLC7A11, which may suppress ferroptosis [46]. The KEGG findings, revealing the downregulation of Hspa5, may indicate a correlation between noise-induced cognitive decline and endoplasmic reticulum dysfunction, highlighting a potential link to the development of Parkinson.

The BP enrichment results of oxidative stress-related DEGs were involved in the regulation of oxidoreductase activity, cellular response to reactive oxygen species, response to reactive oxygen species, cellular response to oxidative stress, and response to hydrogen peroxide. The results of CC enrichment exhibited that the oxidative stress-related DEGs were involved in the inner mitochondrial membrane protein complex, mitochondrial protein-containing complex, mitochondrial respiratory chain complex I, NADH dehydrogenase complex, and respiratory chain complex I. This may highlight that mitochondria and NADPH may be involved in noise-induced cognitive impairment. The results of the KEGG pathway analysis indicated that the oxidative stress-related DEGs were involved in diabetic cardiomyopathy and multiple neurodegenerative diseases, suggesting a correlation between them and chronic noise-induced cognitive impairment.

It is noteworthy that the enrichment analysis was unable to detect the AD pathway. Two potential explanations for this issue exist. Firstly, the sample size was insufficient to identify all ferroptosis-related genes associated with AD. Secondly, ferroptosis is a recently discovered form of cell death, which is one of the reasons why we explored ferroptosis in this study. It is also worth noting that enrichment analyses are often based on pre-existing studies, which may have contributed to the lack of enrichment for AD. This is because the ferroptosis-related genes we screened for in this study may not yet be related to AD in sufficient studies. The evidence presented here does not suggest that noise exposure-induced ferroptosis is unrelated to AD.

Furthermore, PPI networks of these DEGs were constructed, and 16 hub genes were screened (Supplementary Table 4). Hspa5 includes an N-terminal ATPase structural domain, an intermediate ATP-binding structural domain, and a C-terminal protein-binding structural domain. These structural domains enable Hspa5 to interact with other proteins and participate in the protein folding and unfolding processes. It has been demonstrated that Park7 could regulate autophagic flow by binding to Hspa5 [47]. Furthermore, Park7 could inhibit ferroptosis in cerebral ischemia-reperfusion injury via the Atf4/Hspa5 pathway [48]. In Apoe knockout mice, the metabolite Neu5Ac could induce degradation of Slc3a2, resulting in ferroptosis in the vascular endothelium [49]. Besides, Apoe could inhibit ferroptosis by blocking ferritinophagy [50], and the downregulation of Apoe could also be a feature of AD [51]. Transferrin receptor (Tfrc), plays a key role in regulating cellular iron metabolism and maintaining iron homeostasis [52]. Upregulation of Tfrc has also been shown to induce ferroptosis in neuroblastoma [53]. The MR analysis of the correlation between Tfrc and AD revealed significant results of the IVW method (OR = 1.082, 95% CI = 1.012–1.156, *P* = 0.021), MR-Egger’s method (OR = 0.970, 95%CI = 0.776–1.211, *P* = 0.791), and weighted median method (OR = 1.057, 95%CI = 0.965–1.159, *P* = 0.234) (Supplementary Table 1 and Supplementary Fig. 5), suggesting a positive genetic relationship between Tfrc and AD. Taken together, *Hspa5*, *Park7*, *Slc3a2*, and *Apoe*, as protective factors against organismal resistance to ferroptosis, were all downregulated following noise exposure. In contrast, *Tfrc*, a possible risk factor for ferroptosis, was upregulated following noise exposure. This result may suggest that noise exposure plays a contributory role in the development of ferroptosis in the hippocampus. These genes may be targets for the treatment and prevention of chronic noise-induced cognitive impairment.

Additionally, ROC curve analysis was employed to explore the predictive ability of these genes in distinguishing between controls and AD patients, and *Rarres2*, *Tfap2c*, *Klf2*, *Isyna1*, *Eme2*, *Cck*, *Chchd2*, and *Endog* genes all had AUC values greater than 0.7. This suggests that these genes may serve as diagnostic markers for chronic noise-induced cognitive impairment and AD.

*Rarres2* is a ferroptosis-related gene implicated in lipid metabolism reprogramming, and it may regulate circulating serum chemerin [54–56]. Research has shown that *Rarres2* can act as a tumor suppressor gene [57]. Notably, there is a scarcity of research exploring the correlation between *Rarres2* and AD, especially concerning its involvement in ferroptosis. However, the present study utilizing MR analysis revealed a negative genetic association between *Rarres2* and AD. Additionally, bioinformatics analysis revealed that *Rarres2* expression level was downregulated following noise exposure, suggesting that chronic noise may contribute to the development of AD by lowering *Rarres2* expression level.

**Fig. 9:**
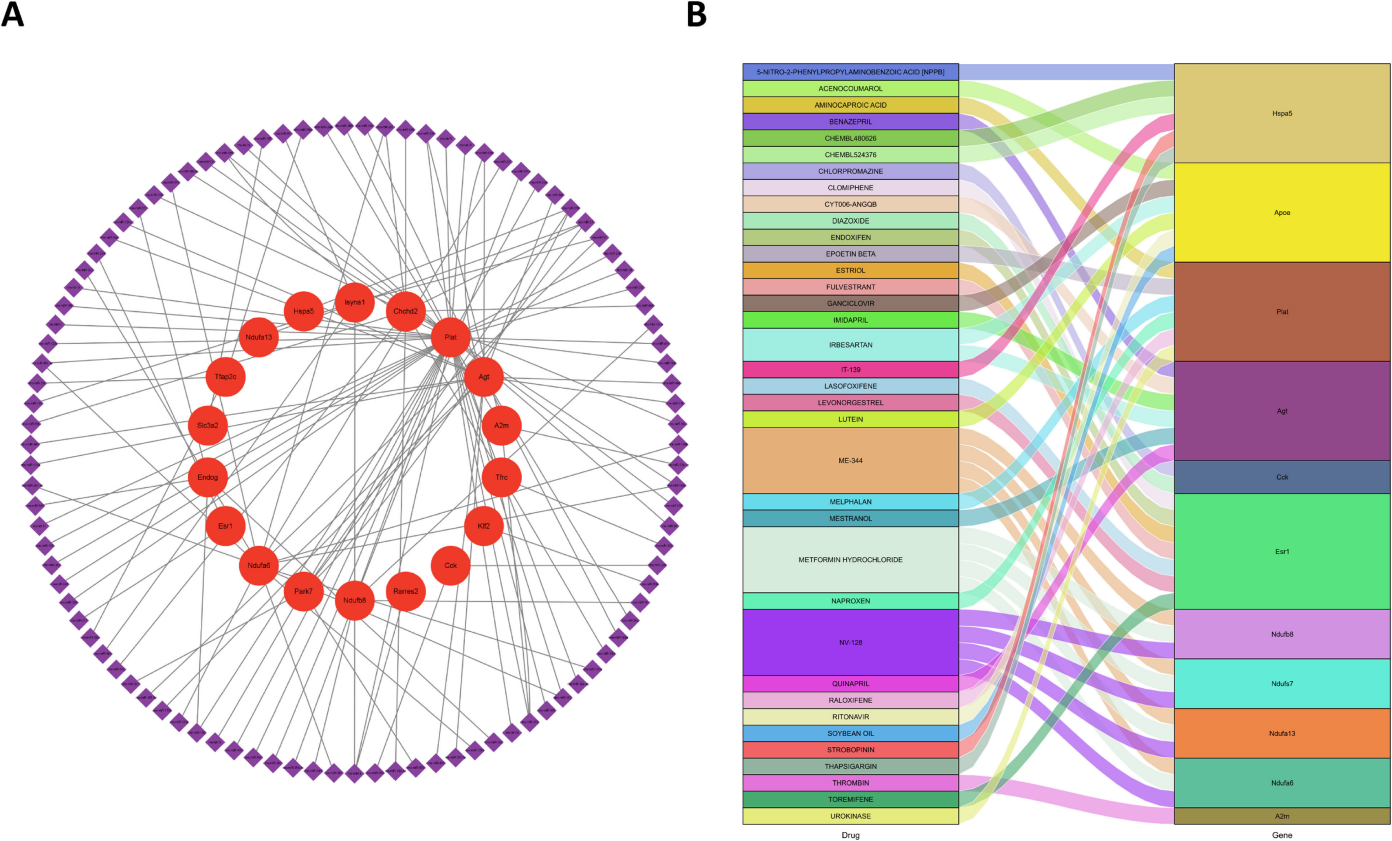
Visualization of gene-miRNA and gene-drug networks. (A) The miRNA-gene regulatory network displays genes as red dots and miRNA as purple squares. (B) The Sankey plot for drug prediction illustrates drugs on the left side and genes on the right side.

In the present study, 12 hub genes, 8 diagnostic-value genes, and 1 gene identified through MR analysis have been obtained. A medication prediction map was constructed using a Sankey diagram and a prediction network of the upstream miRNA of these genes, as they may serve as targets for treating and preventing chronic noise-induced cognitive impairment (Fig. 9). These findings will guide future research into possible mechanisms underlying chronic noise-induced cognitive impairment.

The limitations of this study should be acknowledged. Firstly, the current noise dose settings lack detail. This study was designed to investigate the role of ferroptosis in chronic noise-induced cognitive deficits, so a single dose was used to establish its presence. While the results support our hypothesis, future studies will need to explore varying noise doses, assess changes in ferroptosis levels, and clarify the connection between chronic noise and ferroptosis-related genes. Secondly, although this study implicates ferroptosis as a potential mechanism for chronic noise-induced cognitive impairment, the exact molecular pathways involved were not fully elucidated. Moreover, whether the inhibition of ferroptosis can alleviate chronic noise-induced cognitive impairment remains to be investigated. The relationship among chronic noise exposure, ferroptosis, and cognitive decline may be multifaceted, potentially involving interactions with other cellular processes or pathways not explored in this study. Thirdly, the sample size used for transcriptomics was relatively small, which may result in the omission of some meaningful genes. Although previous studies have demonstrated that three animals per group can be sufficient for transcriptomics analysis, a larger sample size would be beneficial for more comprehensive results [58]. Finally, further large-scale studies focusing on screened central genes and genes of diagnostic value are essential for the more effective prevention and treatment of chronic noise-induced cognitive impairment.

## 5. Conclusions

In summary, this study innovatively examined the roles of ferroptosis and Rarres2 in chronic noise-induced cognitive impairment. Moreover, oxidative stress- and ferroptosis-related DEGs were identified through bioinformatics analysis, followed by functional enrichment analysis, PPI network analysis, ROC curve analysis, and MR analysis. Genes that have diagnostic value or are causally linked to AD may be targets for prevention and treatment of chronic noise-induced cognitive impairment and AD.

## Abbreviations

MR: Mendelian randomization
AD: Alzheimer’s disease
WHO: World Health Organization
ROS: Reactive oxygen species
Rarres2: Retinoic Acid Receptor Responder 2
SNPs: Single nucleotide polymorphisms
WB: Western Blot
MWM: Morris water maze
MDA: Malondialdehyde
SOD: Superoxide Dismutase
GSH: Glutathione
GSH-Px: Glutathione Peroxidase
DEGs: Differentially expressed genes
GO: Gene Ontology
KEGG: Kyoto Encyclopedia of Genes and Genomes
GSEA: Gene Set Enrichment Analysis
TF: Transcription factors
GEO: Gene Expression Omnibus
CON: Control
NE: Noise Expose
ROC: Receiver Operating Characteristic
AUC: An area under the curve
OR: Odds ratio
CI: confidence interval
AAALAC: Association for Assessment and Accreditation of Laboratory Animal Care International
IACUC: Institutional Animal Care and Use Committee

## Supplementary information

**Additional file 1:** **Supplementary Figure 1** The study flowchart. **Supplementary Figure 2** The enrichment pathway analysis of oxidative stress-related hub genes and ferroptosis-related hub genes. **Supplementary Figure 3** The visualization of the MR study on the relationship between two ferroptosis-related genes and AD. **Supplementary Figure 4** Visualization of the MR analysis of Tfrc on AD. **Supplementary Figure 5** Visualization of the reverse MR analysis of AD on ferroptosis-related genes. **Supplementary Table 1** Mendelian randomization estimates for the associations between Ferroptosis-related genes and AD. **Supplementary Table 2** Heterogeneity of Wald ratios for the associations between Ferroptosis-related genes and AD. **Supplementary Table 3** MR-Egger’s analysis of directional pleiotropy for the associations between Ferroptosis-related genes and AD. **Supplementary Table 4** The classification of hub genes.

## Declarations

### Availability of data and material

This study has no associated data.
