# Supplementary material for: Chronic noise exposure induces Alzheimer’s disease-like neuropathology and cognitive impairment via ferroptosis in rat hippocampus
Source: Environ Health Prev Med. 2024 Sep 28;29:50. doi: 10.1265/ehpm.24-00126 (PMC11446637; doi:10.1265/ehpm.24-00126)
Supplement: Supplementary file 1 — Additional file 1: Supplementary Figure 1 The study flowchart. Supplementary Figure 2 The enrichment pathway analysis of oxidative stress-related hub genes and ferroptosis-related hub genes. Supplementary Figure 3 The visualization of the MR study on the relationship between two ferroptosis-related genes and AD. Supplementary Figure 4 Visualization of the MR analysis of Tfrc on AD. Supplementary Figure 5 Visualization of the reverse MR analysis of AD on ferroptosis-related genes. Supplementary Table 1 Mendelian randomization estimates for the associations between Ferroptosis-related genes and AD. Supplementary Table 2 Heterogeneity of Wald ratios for the associations between Ferroptosis-related genes and AD. Supplementary Table 3 MR-Egger’s analysis of directional pleiotropy for the associations between Ferroptosis-related genes and AD. Supplementary Table 4 The classification of hub genes. [file ehpm-29-050-s001.docx]

Supplementary Materials for

**Chronic noise exposure induces Alzheimer’s disease-like neuropathology and cognitive impairment via ferroptosis in rat hippocampus**

**This file includes:**

Supplementary figure 1-5

Supplementary table 1-4

**Supplementary figure**


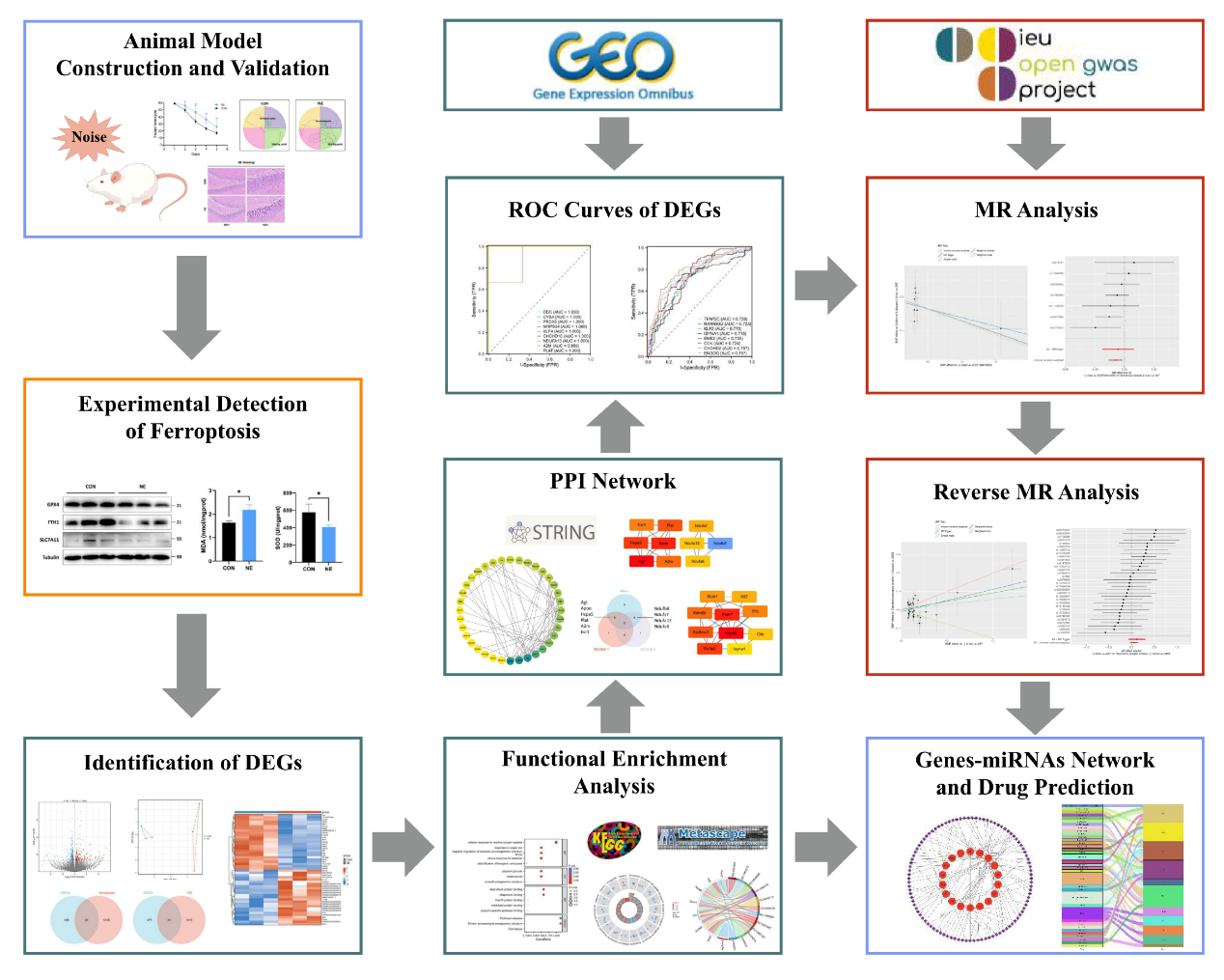


**Supplementary Figure 1** The study flowchart.


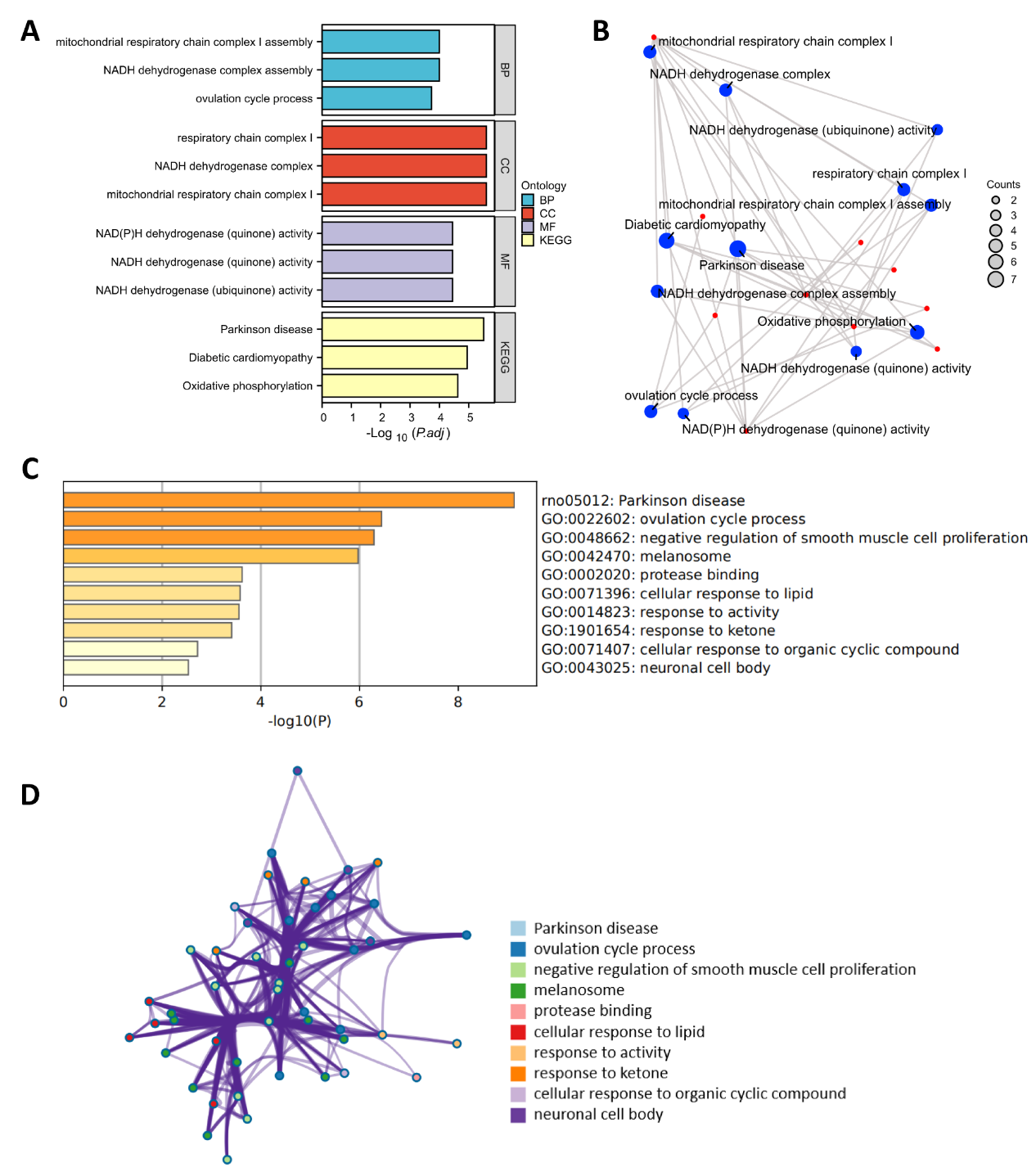


**Supplementary Figure 2** The enrichment pathway analysis of oxidative stress-related hub genes and ferroptosis-related hub genes. (A) The results of GO and KEGG functional enrichment analyses of these 12 genes (B) The network of the enriched pathways. (C) The enrichment analysis of these 12 genes obtained by Metascape. (D) The pathway network of these 12 genes obtained using Metascape.


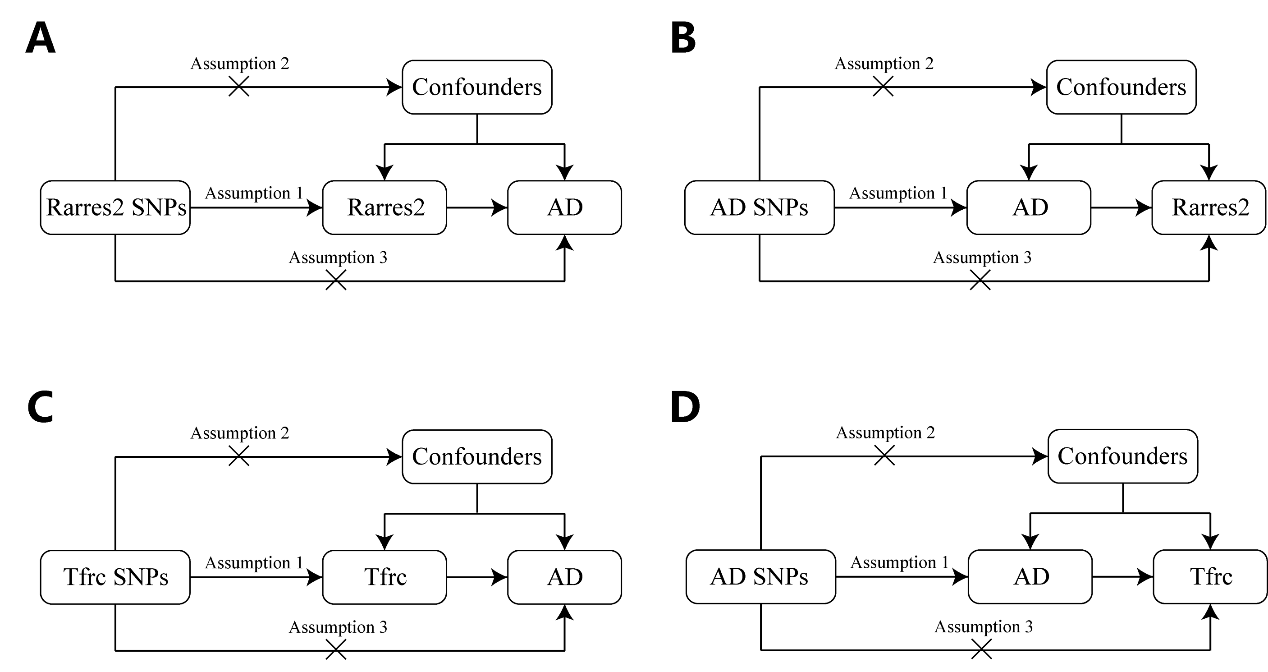


**Supplementary Figure 3** The visualization of the MR study on the relationship between two ferroptosis-related genes and AD. (A) Rarres2 SNPs as IVs to explore the causal relationship between Rarres2 and AD. (B) AD SNPs as IVs to explore the causal relationship between Rarres2 and AD. (C) Tfrc SNPs as IVs to explore the causal relationship between Tfrc and AD. (D) AD SNPs as IVs to explore the causal relationship between Tfrc and AD.
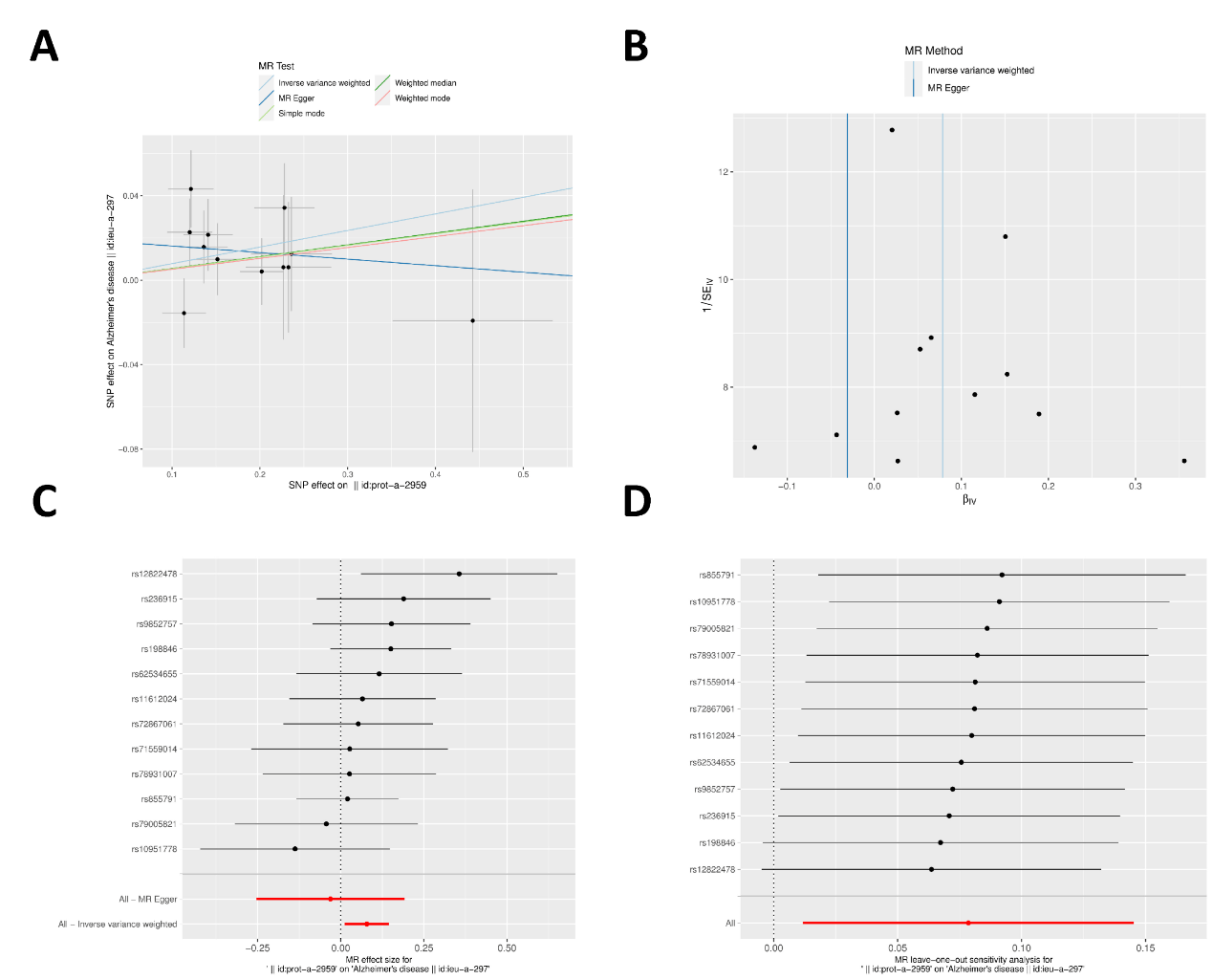


**Supplementary Figure 4**  Visualization of the MR analysis of Tfrc on AD. (A) The SNP effect sizes for Tfrc and AD. (B) The funnel plot illustrating no significant heterogeneity among IVs of Tfrc. (C) The forest plots of causal effects of Tfrc SNPs on AD. (D) The leave-one-out analysis of the effect of Tfrc SNPs on AD.


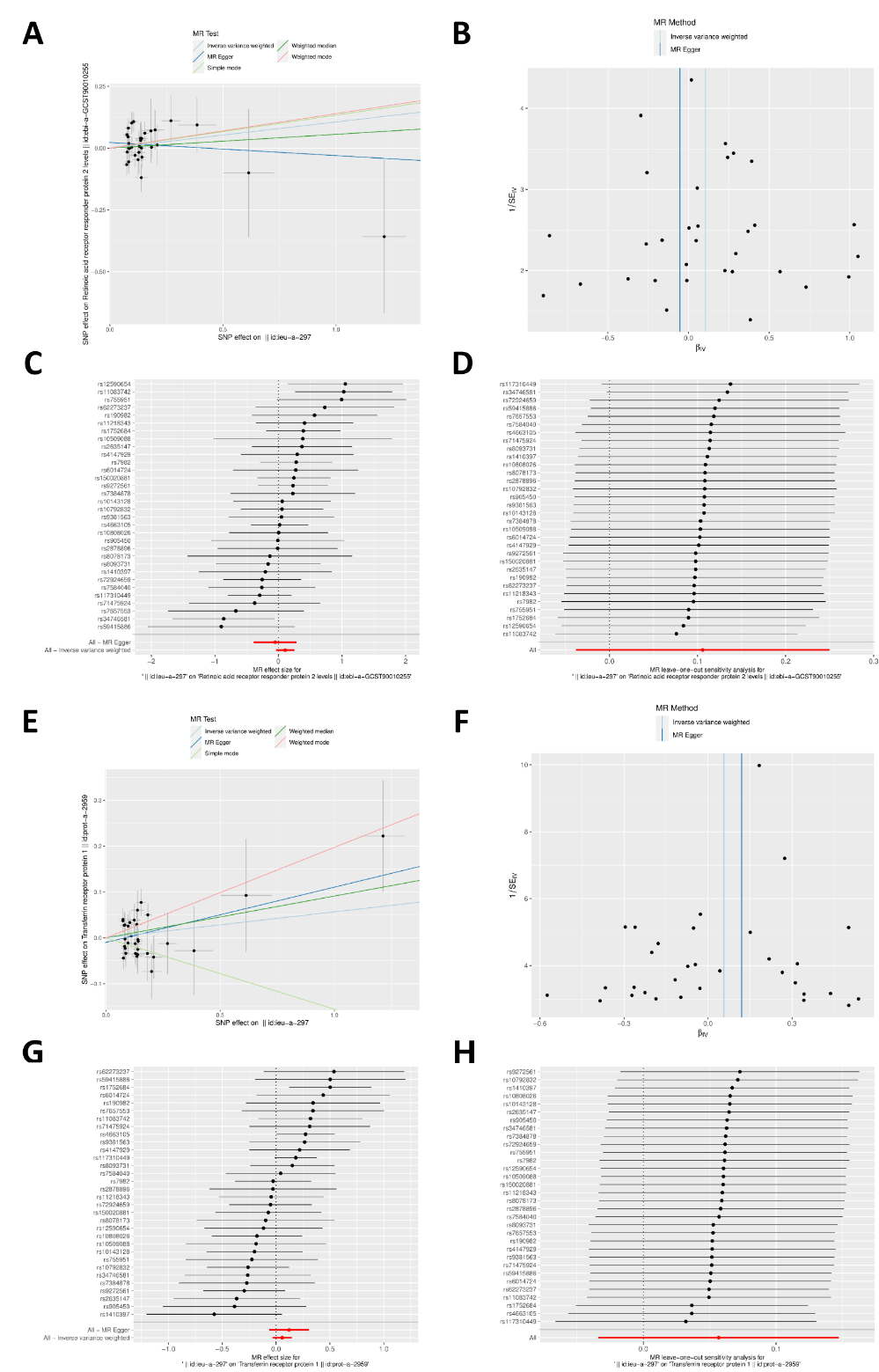


**Supplementary Figure 5** Visualization of the reverse MR analysis of AD on ferroptosis-related genes. (A, E) The SNP effect sizes for AD and ferroptosis-related genes. (B, F) The funnel plot displaying no significant heterogeneity among IVs of AD. (C, G) The forest plots of causal effects of AD SNPs on ferroptosis-related genes. (D, H) The leave-one-out analysis of the effect of AD SNPs on ferroptosis-related genes.

**Supplementary tables**

**Supplementary Table 1** Mendelian randomization estimates for the associations between Ferroptosis-related genes and AD

| **Exposure** | **Outcome** | **Method** | **OR** | **95%CI** | **P value** |
| --- | --- | --- | --- | --- | --- |
| Rarres2 | AD | MR Egger | 0.950 | 0.831-1.086 | 0.485 |
|  |  | Weighted median | 0.931 | 0.862-1.006 | 0.072 |
|  |  | Inverse variance weighted | 0.928 | 0.876-0.984 | 0.012 |
|  |  | Simple mode | 0.932 | 0.831-1.045 | 0.272 |
|  |  | Weighted mode | 0.932 | 0.853-1.018 | 0.168 |
| AD | Rarres2 | MR Egger | 0.760 | 0.678-1.327 | 0.760 |
|  |  | Weighted median | 0.572 | 0.871-1.283 | 0.572 |
|  |  | Inverse variance weighted | 0.150 | 0.962-1.284 | 0.150 |
|  |  | Simple mode | 0.492 | 0.785-1.664 | 0.492 |
|  |  | Weighted mode | 0.380 | 0.846-1.562 | 0.380 |
| Tfrc | AD | MR Egger | 0.969 | 0.776-1.212 | 0.791 |
|  |  | Weighted median | 1.057 | 0.965-1.159 | 0.234 |
|  |  | Inverse variance weighted | 1.082 | 1.012-1.156 | 0.021 |
|  |  | Simple mode | 1.057 | 0.922-1.211 | 0.445 |
|  |  | Weighted mode | 1.053 | 0.921-1.204 | 0.466 |
| AD | Tfrc | MR Egger | 1.128 | 0.938-1.356 | 0.210 |
|  |  | Weighted median | 1.095 | 0.963-1.247 | 0.167 |
|  |  | Inverse variance weighted | 1.058 | 0.967-1.159 | 0.220 |
|  |  | Simple mode | 0.856 | 0.631-1.161 | 0.325 |
|  |  | Weighted mode | 1.218 | 1.010-1.469 | 0.048 |

**Supplementary Table 2** Heterogeneity of Wald ratios for the associations between Ferroptosis-related genes and AD

| **Exposure** | **Outcome** | **Q** | **df** | **P** |
| --- | --- | --- | --- | --- |
| Rarres2 | AD | 6.003 | 6 | 0.423 |
| AD | Rarres2 | 35.112 | 31 | 0.279 |
| Tfrc | AD | 8.985 | 11 | 0.623 |
| AD | Tfrc | 40.311 | 31 | 0.122 |

**Supplementary Table 3** MR-Egger’s analysis of directional pleiotropy for the associations between Ferroptosis-related genes and AD

| **Exposure** | **Outcome** | **Intercept** | **SE** | **P** |
| --- | --- | --- | --- | --- |
| Rarres2 | AD | -0.010 | 0.026 | 0.718 |
| AD | Rarres2 | 0.023 | 0.022 | 0.314 |
| Tfrc | AD | 0.019 | 0.019 | 0.337 |
| AD | Tfrc | -0.010 | 0.013 | 0.441 |

**Supplementary Table 4** The classification of hub genes.

| **Gene classification** | **Down****regulated gene list** | **Upregulated gene list** |
| --- | --- | --- |
| Ferroptosis-related hub genes | Hspa5, Park7, Slc3a2, Atp5mc3, Kdm6b | Tfrc |
| oxidative stress-related hub genes | Hspa5, Apoe, Agt, Plat, A2m, Ndufa6, Ndufa7, Ndufa13 | Esr1, Ndufa8 |
| Share genes | Hspa5 | — |
